# Effects of Kinect-based exergaming on frailty status and physical performance in prefrail and frail elderly: A randomized controlled trial

**DOI:** 10.1038/s41598-019-45767-y

**Published:** 2019-06-27

**Authors:** Ying-Yi Liao, I.-Hsuan Chen, Ray-Yau Wang

**Affiliations:** 1 Department of Gerontological Health Care, National Taipei University of Nursing and Health Sciences, Taipei, Taiwan; 2 Department of Physical Therapy, Fooyin University, Kaohsiung, Taiwan; 3 Department of Physical Therapy and Assistive Technology, National Yang-Ming University, Taipei, Taiwan

**Keywords:** Rehabilitation, Geriatrics

## Abstract

Frailty status can be improved by intervention. Both exergaming and combined exercise have been proposed for improving physical performance in community-dwelling elderly. However, whether frailty status can be improved by exergaming is unclear. Moreover, whether Kinect-based exergaming training can exert a stronger effect on improving frailty status than combined exercise needs to be established. The aim of this study was to investigate the effects of Kinect-based exergaming on improving frailty status and physical performance in the prefrail and frail elderly by comparing its effects with those of combined exercise. Fifty-two prefrail and frail elderly were recruited and randomized to the Kinect-based exergaming group (EXER group) or combined exercise group (CE group), emphasizing resistance, aerobic, and balance training for 36 sessions over 12 weeks. Our results showed that both groups improved the frailty status (EXER group: p = 0.016, effect size = 2.29; and CE group: p = 0.031, effect size = 2.67). Three out of 5 physical characteristics of the frailty phenotype, namely, weakness, slow walking speed, and low activity level, were significantly reversed by both exergaming and combined exercise. However, the exergaming training also significantly reversed exhaustion. Furthermore, compared with the CE group, the EXER group showed greater improvement in dynamic balance control, as indicated by the forward reaching test (p = 0.0013, effect size = 0.40) and single leg stance test (p = 0.049, effect size = 0.42). Thus, Kinect-based exergaming exerted effects that were at least as beneficial as those of combined exercise in improving frailty status and the frailty phenotype. We recommend the use of exergaming aided by Kinect in the prefrail and frail elderly.

## Introduction

“Frailty” is an aging syndrome between normal aging and dependence. Frailty is diagnosed when the functions of multiple organs in the body gradually decline, and the biological reserve falls below a certain level^1^. There are two main definitions of the frailty phenotype. One definition is characterized by a multi-domain phenotype including physical, cognitive, psychological, and social deficits^2^. The other definition, based on Fried’s classification, focuses on the physical phenotype and lists five physical characteristics of the frailty phenotype, namely, unintentional weight loss, muscle weakness, exhaustion, slow walking speed, and a low level of physical activity^2^. Physical exercise has been shown to improve physical performance and prevent the progression of physical frailty^3,4^. Therefore, we choose Fried’s frailty phenotype definition as our outcome to compare the effects of the different interventions on physical frailty prevention. According to this definition, the presence of 1-2 characteristics is defined as prefrailty, and the presence of 3–5 characteristics is defined as frailty^5^. According to a systematic review, the prevalence of prefrailty in the community-dwelling older population ranges from 38–53%, and the prevalence of frailty ranges from 4–17%^6^. In Taiwan, the prevalence of frailty is 11% to 14.9% in community-dwelling older adults^7^.

Frail older adults have a high risk of becoming physically dependent; however, their frailty status can be improved^8,9^. Therefore, strategies to improve physical performance and frailty status are important. Exercise is one evidence-based intervention that can be applied to delay functional dependence and improve physical functioning in the elderly. Aerobic, resistance, balance, and flexibility exercises have been recommended for older people^8^. Combined exercise, composed of multiple types of exercises, is feasible for older people to engage in to achieve health benefits^9^. Evidence has shown that combined exercise can improve gait and balance, increase muscle strength, and reduce fall risks among community-dwelling older adults^10–12^. However, few studies have assessed the effect of combined exercise on frailty prevention as the main outcome measure.

Exergaming is the process of playing a computer game that is controlled by body movements and that provides augmented feedback in real time while a person performs specific cognitive and motor tasks. Moreover, exergaming is effective at retaining participants’ interest and motivation^13^. Therefore, this new type of intervention has been proposed as a complementary tool for improving the health of the elderly.

Kinect is a recently developed exergaming device that has the advantage of low cost. In addition, Kinect can capture the joint movements of the whole body and is thus similar to virtual reality in its ability to allow the user to interact directly with a computer-simulated environment, unlike a Wii exergaming device^14^. Previous findings suggested that Kinect could be a useful tool for therapy^15^. Evidence showed that Kinect-based exergaming training can improve single leg standing and functional abilities in community-dwelling elderly^16^. However, studies about the effects of Kinect-based exergaming on physical performance and frailty status in prefrail and frail elderly are still lacking. Moreover, whether Kinect-based exergaming training can exert a stronger effect than combined exercise needs to be established for future applications.

Therefore, the purpose of this study was to investigate the effects of Kinect-based exergaming and combined exercise training on improving the frailty status and physical performance of the prefrail and frail elderly.

## Results

**Figure 1:**
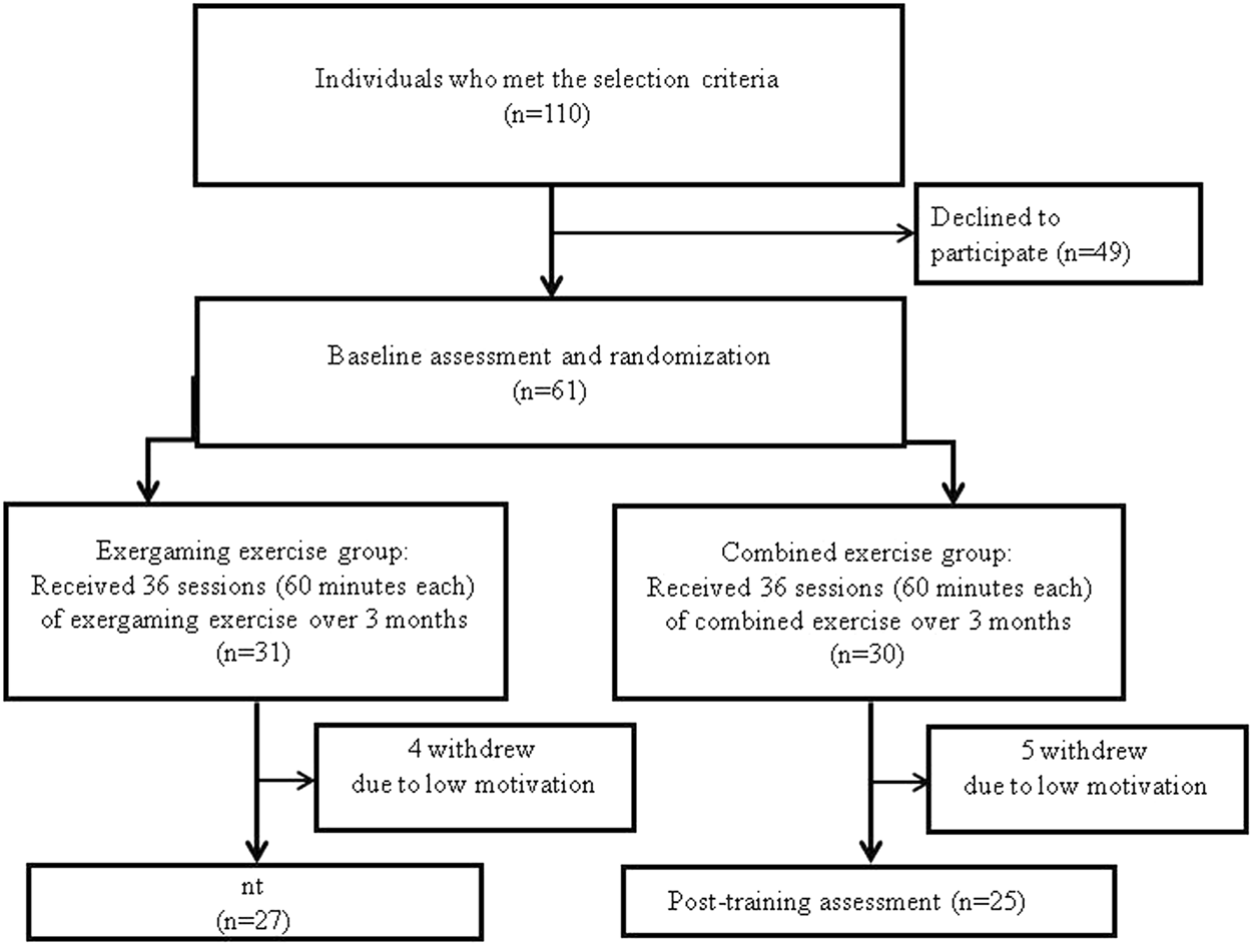
Flowchart of this study.

As shown in the flowchart (Fig. 1), 110 individuals were initially screened for participation in this study. Among them, 61 qualified individuals were randomly assigned to either the exergaming group (EXER group, 31 participants) or the combined exercise group (CE group, 30 participants). Of the 61 participants, nine did not complete the study due to low levels of motivation (4 in the EXER group and 5 in the CE group). Therefore, a total of 52 participants (27 in the EXER group and 25 in the CE group) completed all the interventions and assessments. None of the participants reported any adverse events.

The demographic characteristics of the participants are presented in Table 1. There were no significant differences in the demographic parameters of the two groups. Moreover, there was no significant difference between the two groups regarding all the outcome measures during the pre-intervention assessments (Tables 2 and 3).

**Table 1:** Baseline demographic characteristics of the participants (N = 52).

	Exergaming group	Combined exercise group	P-value
	(N = 27)	(N = 25)	
Age (years)	79.6 ± 8.5	84.1 ± 5.5	0.068
Gender (female/male)	19/8	17/8	0.853
Height (cm)	156 ± 5.2	159 ± 10.5	0.415
Body weight (kg)	52.6 ± 6.0	57.5 ± 8.8	0.185
BMI	21.5 ± 2.2	22.5 ± 2.4	0.331
Fall history in the past one year	8	9	0.653
Frailty score	3.0 ± 1.3	3.0 ± 1.4	0.994
Frailty status			
Prefrail/frail	11/16	9/16	0.726
Frailty phenotype (%)			
Weakness	20 (74.1%)	21 (84.0%)	0.381
Slow walking speed	21 (77.8%)	19 (76.0%)	0.879
Unintentional weight loss	3 (11.1%)	5 (20.0%)	0.375
Exhaustion	20 (74.1%)	14 (56.0%)	0.171
Low activity level	18 (66.7%)	15 (60.0%)	0.618

Data are presented as the mean ± SD or number.

**Table 2:** Comparisons of frailty scores, frailty status, reversal rates, and frailty phenotype (N = 52).

	Exergaming group (N = 27)				Combined exercise group (N = 25)				Between-group difference, P
	Pre-intervention	Post-intervention		Within-group difference, P	Pre-intervention	Post-intervention		Within-group difference, P	
Frailty score	3.0 ± 1.3	1.8 ± 1.5		<0.001	3.0 ± 1.4	2.1 ± 1.3		<0.001	0.075
Frailty status (%)									
Frailty elderly	16 (59.3%)	9 (33.3%)			16 (64.0%)	10 (40.0%)			0.442
Prefrail elderly	11 (40.7%)	10 (37.0%)			9 (36.0%)	11 (44.0%)			
Robust elderly	0 (0%)	8 (29.6%)			0 (0%)	4 (16.0%)			
Frailty reverse rate		43.8%		0.016		37.5%		0.031	0.262
Frailty phenotype (%)			**Reversal rate**				**Reversal rate**		
Weakness	20 (74.1%)	12 (44.4%)	40%	0.008	21 (84.0%)	13 (52.0%)	38.1%	0.008	0.901
Slow walking speed	21 (77.8%)	11 (40.7%)	47.6%	0.002	19 (76.7%)	13 (52.0%)	31.5%	0.031	0.301
Unintentional weight loss	3 (11.1%)	3 (11.1%)	0%	1.000	5 (17.2%)	3 (10.0%)	40%	0.500	0.464
Exhaustion	20 (74.1%)	10 (37.0%)	50%	0.002	14 (53.8%)	11 (40.0%)	28.6%	0.25	0.092
Low activity level	18 (66.7%)	10 (37.0%)	44.4%	0.008	15 (60.0%)	8 (32.0%)	46.7%	0.016	0.898

Data are presented as the mean ± SD.

**Table 3:** Comparisons of physical performance and FES-I.

	Exergaming group (N = 27)			Combined exercise group (N = 25)			Time × group, P
	Pre-intervention	Post-intervention	Time effect, P	Pre-intervention	Post-intervention	Time effect, P	
Physical performance							
Back scratch (cm)	15.4 ± 18.9	15.3 ± 16.5	0.980	19.0 ± 7.6	16.6 ± 10.4	0.357	0.654
Chair sit and reach (cm)	4.3 ± 8.3	3.0 ± 10.7	0.594	9.3 ± 6.5	5.5 ± 4.7	0.206	0.852
Thirty-second sit-to-stand test (times)	9.9 ± 5.1	13.0 ± 5.8	<0.001	8.9 ± 4.9	11.8 ± 5.4	<0.001	0.824
Functional reach test (cm)	20.3 ± 8.2	25.9 ± 9.1	<0.001	22.7 ± 5.49	24.2 ± 7.1	0.316	0.013
Single leg stance (seconds)	3.9 ± 3.6	9.4 ± 8.2	0.028	5.4 ± 7.7	5.1 ± 3.7	0.676	0.049
Stepping (steps)	75.2 ± 29.4	74.2 ± 29.5	0.886	97.8 ± 25.4	86.6 ± 30.6	0.258	0.928
Timed up and go (seconds)	17.0 ± 8.4	15.1 ± 8.7	0.005	16.6 ± 9.9	15.4 ± 8.2	0.196	0.668
Grip strength (kg)	15.4 ± 5.0	18.2 ± 5.4	<0.001	13.7 ± 5.5	17.0 ± 5.6	<0.001	0.563
Walking velocity (m/s)	0.61 ± 0.33	0.74 ± 0.29	0.001	0.62 ± 0.24	0.68 ± 0.27	0.043	0.215
Fall efficacy scale (FES-I)	44.0 ± 10.1	37.3 ± 9.5	<0.001	40.2 ± 8.2	35.7 ± 7.4	<0.001	0.084

Data are presented as the mean ± SD.

### Primary outcomes

The results of the primary outcomes are shown in Table 2. No significant differences between groups were observed in frailty scores (p = 0.075). Furthermore, there was no significant group effect on the frailty reversal rate (p = 0.262) or the frailty phenotype reversal rate (p = 0.091–0.899).

However, significant within-group effects were noted for the frailty scores (p < 0.001, effect size = 1.7) and frailty reversal rate (p = 0.016, effect size = 2.29) in the EXER group. Seven of the 16 frail elderly (43.7%) improved to a prefrail status, and 8 of the 11 prefrail elderly (72.7%) improved to a robust status. The reversal rates of the characteristics of the frailty phenotype were also significant after exergaming exercise, except for unintentional weight loss (p = 0.008, effect size = 2.50 for weakness; p = 0.002, effect size = 2.10 for slow walking speed; p = 0.002, effect size = 2.00 for exhaustion; p = 0.008, effect size = 2.25 for low activity level). For the CE group, significant within-group effects were noted for the frailty scores (p < 0.001.effect size = 1.37) and frailty reversal rate (p = 0.031, effect size = 2.67). Six of the 16 frail elderly (37.5%) improved to a prefrail status, and 4 of the 9 prefrail elderly (44.4%) improved to a robust status. The reversal rates of the characteristics of the frailty phenotype were significant after CE training for weakness (p = 0.008, effect size = 2.63), slow walking speed (p = 0.031, effect size = 2.10), and low activity level (p = 0.016, effect size = 2.14).

The results of the secondary outcomes are shown in Table 3. Significant within-group effects were noted for the 30-second sit-to-stand (p < 0.001, effect size = 1.76), single leg stance (p = 0.028, effect size = 1.02), functional reach (p < 0.001, effect size = 1.36), timed up and go (p = 0.005, effect size = 0.67), walking speed (p = 0.001, effect size = 0.59), grip strength (p < 0.001, effect size = 0.89), and FES-I (p < 0.001, effect size = 1.88) tests in the EXER group. Significant within-group effects were noted for the 30-second sit-to-stand (p < 0.001, effect size = 1.37), walking speed (p = 0.043, effect size = 0.64), grip strength (p < 0.001, effect size = 1.37), and FES-I (p < 0.001, effect size = 1.05) tests in the CE group. The analysis also showed significantly greater improvement in the single leg stance (p = 0.0049, effect size = 0.42) and functional reach (p = 0.013, effect size = 0.40) tests after 12 weeks of EXER training than after CE training.

## Discussion

In the present study, we found significant improvements in the frailty scores and statuses, along with improvements in physical performance with Kinect-based exergaming for the prefrail and frail older adults. According to a systematic review, frail elderly benefit from exercise interventions^17^. Most studies have focused on the effect of exercise on improving physical performance, while few studies have investigated the effect on frailty status and elements of frailty phenotypes. Separating the elements of frailty is important for understanding the effect of the intervention on individual components of the frailty phenotype instead of the overall frailty status. Frailty trajectories have shown that older adults change their status naturally without any mediating intervention. However, frailty status can be improved through interventions. Francisco *et al*. reported that frailty scores were reversed from 3.6 to 1.6 after 6 months of multicomponent exercises, while the frailty scores control group (without any intervention) remained unchanged (3.8) in the frail elderly. Kim *et al*.^18^ also reported that exercise can decrease frailty scores after 3 months of traditional intervention in community-dwelling frail women. Although our study period is not long enough to compare the natural trajectories of frailty, the improved frailty scores in our EXER group (3.0 to 1.8) and CE group (3.0 to 2.1) proved that frailty status can be improved in a short time period (3 months) through intensive interventions. In the present study, a significant improvement in frailty scores and in the frailty reversal rates (43.75% frail elderly reverted to a prefrail status, and 72.7% prefrail elderly reverted to a robust status) were noted after Kinect-based exergaming training. These results provide clinical evidence about using this innovative intervention in prefrail and frail populations.

Fried described five physical characteristics of the frailty phenotype, namely, unintentional weight loss, weakness, exhaustion, slow gait speed, and a low physical activity level. In the present study, 4 out of these 5 physical characteristics of the frailty phenotype, namely, weakness, exhaustion, slow gait, and low physical activity level were significantly reversed after exergaming training. Among those 4 physical characteristics, exhaustion was not significantly reversed after combined exercise.

Weak grip strength in later life is a risk factor for health outcomes, including higher disability, mortality and morbidity^19,20^. Grip strength testing is increasingly used in clinical settings for the assessment of sarcopenia and frailty in elderly people^21^. Kim *et al*. found no significant improvement in grip strength after a traditional exercise intervention^18^. However, different results were noted in the present study. In our program, we included many upper extremity resistance exercises, such as TheraBand exercises and catching, squeezing and resisting a rubber ball in both the EXER and CE groups, while most of the programs in the other studies focused on lower extremity strengthening exercises^22^. Training-induced gains through resistance training may lead to increases in the mass and intrinsic contractile characteristics of muscles. In our results, both the EXER and CE groups demonstrated similar training effects on grip strength. Therefore, upper extremity resistance training should be incorporated to improve grip strength and possibly general health in the elderly population.

Gait speed is significantly associated with transitions in an individual’s frailty status^23^. Slow gait speed is the strongest predictor of chronic disability and injurious falls^24,25^. According to our results, gait speed improved by 0.13 m/s after exergaming training and by 0.06 m/s after combined exercise. The clinically significant change in gait speed is 0.05 m/s in older adults^26^. Therefore, improvements in gait speed in both groups were clinically important. Older adults are more likely to adhere to a physical activity program if the program is well designed and enjoyable^27^. The improvement in low activity levels implies that our programs, both exergaming and CE, may motivate participants to spend more time doing daily activities, which might prevent a sedentary lifestyle. Exhaustion is a key physical characteristic of Fried’s frailty phenotype. There are a number of physical and psychological causes of exhaustion, including sleep apnea, depression, hypotension, and B12 deficiency. From the results of this self-reported questionnaire, exergaming training programs significantly reversed exhaustion, which further indicates that this interactive and motivating program can improve self-reported well-being.

A previous systematic review stated that multifaceted (exercise combined with nutrition such as protein supplementation) intervention is more efficient than mono-domain (exercise alone) intervention in improving muscle mass^28,29^ and reversing unintentional weight loss^30^. According to the Asia Pacific clinical practice guidelines for the management of frailty, protein and caloric supplementation is conditionally recommended for weight loss^31^. Therefore, the nonsignificant reversal rate of unintentional weight loss in our present study may be due to the emphasis on mono-domain exercise training instead of a multifaceted intervention.

In this study, we demonstrated that Kinect-based exergaming training can exert at least the same effects on improving frailty scores and reversing frailty status as combined exercise training in the prefrail and frail elderly. In addition, the dynamic balance abilities indicated by the forward reach distance and one leg stance duration were significantly better after exergaming training than after the combined training. Recently, the relationships between frailty and dynamic balance have been established, indicating that impaired balancing ability may contribute to frailty^32,33^. Dynamic standing balance tasks are used to discriminate between frail, prefrail, and robust elderly^34^. Dynamic balance is also an important indicator for fall risk. It is reported that a high fall risk threshold for single leg stance is 6 seconds and the recurrent fall risk criterion for forward reaching distance is 25.4 cm^35–37^. In the EXER group, the average single leg stance duration improved from 3.9 to 9.4 seconds and the forward reach distance improved from 20.3 to 25.9 cm. After exergaming, six more subjects (from 4 to 10) passed the high fall risk threshold for single leg stance and 6 more subjects (from 8 to 14) passed the recurrent fall risk criterion for forward reaching distance. However, the number of subjects that passed the high fall risk threshold didn’t increase compared to the baseline, and only 2 more subjects (from 7 to 9) passed the recurrent fall risk criterion after control training. These results indicate that exergaming training improves balance performance and reduces possible fall risk.

The significantly greater improvement in dynamic balance after exergaming training than after combined training may further support the advantages of exergaming. It is known that Kinect-based exercise gaming is enjoyable and competitive Participants were then able to make adjustments according to real-time auditory and visual feedback, thus enhancing their skills and motor performance. The gaming programs can also be combined with physical and cognitive demands in an attractive and interactive way. Therefore, we speculate that the cognitive function of participants after exergaming training may be improved in addition to their physical performance improvement.

A previous study stated that Wii-based exergaming significantly improved lower extremity strength and balance in community-dwelling elderly^38^. In this study, prefrail and frail elderly also benefited from Kinect-based exergaming exercise. The lower extremity muscle strength, endurance, agility, dynamic balance, walking speed, and grip strength were all significantly improved after exergaming training according to our results. These improvements may extend to reducing the participants’ concerns about falling, as indicated by the FES-I score in the present study. Furthermore, FES-I not only assesses basic daily activities at home but also includes measurements of social activities outside the home, such as walking in a crowd, on slippery and uneven surfaces, as well as up and down a slope. Taken together, the Kinect-based exergaming training can be a choice of training for the prefrail and frail elderly to improve their frailty status and physical performance, and possibly decrease the risk of falling risks.

There are several limitations to this study. First, the lack of follow-up assessment after 12 weeks of intervention limits the inference of the long-term or maintenance effect of the treatment. Second, our participants were recruited from elderly care centers. Regular physical and cognitive training were provided in some centers, which may confound the intervention effect. Third, some important variables such as comorbidities and depression were not assessed in this study, which may affect our results. Fourth, marginal age differences were noted between the groups although these differences were not statistically significant. The relatively younger EXER group may benefit from the treatment more compared to the relatively older CE group. Finally, different frailty populations were defined by different frailty criteria. Thus, the results for our populations, defined by the Fried criteria, may not be generalizable to populations defined by other frailty tools.

## Conclusion

Our results demonstrated that Kinect-based exergaming is at least as beneficial as combined exercise in the prefrail and frail elderly. A 12-week Kinect-based exergaming program effectively improves frailty status and frailty characteristics in the prefrail and frail elderly, and such effects may be due to improvements in physical performance. These findings support the potential therapeutic use of exergaming aided by Kinect for the prefrail and frail elderly.

## Methods

### Participants

This study protocol was approved by the Institutional Human Research Ethics Committee of National Yang-Ming University. The approved protocol was followed throughout the study period. This trial was registered at http://www.clinicaltrials.in.th/ (TCTR20180531001 on 24-May-2018) and conformed to the CONSORT checklist(supplementary information). Participants were recruited from daycare centers for the elderly in Taiwan. All participants met the following inclusion criteria: (1) age between 65 to 90 years old and (2) the presence of at least one of the 5 following physical characteristics defined by Fried: unintentional body weight loss, exhaustion, weakness, slow gait speed, and low physical activity level^5^.

1. Unintentional body weight loss was defined as having unintentionally lost more than 1.5 kg in the past 3 months. In the present study, weight loss of 4.5 kg was modified to 1.5 kg because our intervention duration was only 3 months.
2. Exhaustion was defined as the participant agreeing with one of the following two statements: “I felt too tired to do anything” or “I lacked the motivation to do anything”, for at least 3 days in the past week.
3. Weakness was defined as the grip strength of the dominant hand (mean of three measurements) using a hand-held dynamometer. The cutoff grip strengths were as follows: for males with BMIs ≤ 24, grip strength ≤ 29 kg; for males with BMIs 24.1–28, grip strength ≤ 30 kg; for males with BMIs > 28, grip strength ≤ 32 kg; for females with BMIs ≤ 23, grip strength ≤ 17 kg; for females with BMIs 23.1–26, grip strength ≤ 17.3 kg; for females with BMIs 26.1–29, grip strength ≤ 18 kg; for females with BMIs > 29, grip strength ≤ 21 kg.
4. Slow walking speed was defined by a cutoff value for the time it took the participant to walk 4.57 m at a usual pace. For males taller than 173 cm, the cutoff time was 6 seconds; for males shorter than 173 cm, the cutoff time was 7 seconds; for females shorter than 159 cm, the cutoff time was 7 seconds; and for females taller than 159 cm, the cutoff time was 6 seconds.
5. A low physical activity level was defined as burning fewer than 594 kcal per week for men and 295 kcal per week for women, and the calories burned were calculated according to the Taiwan short form of the International Physical Activity Questionnaire (IPAQ) modified for the elderly.

The exclusion criteria were as follows: (1) the presence of unstable medical conditions, such as neurological or cardiopulmonary diseases, that may interfere with participation in the exercise intervention and (2) severe visual impairment that may interfere with the exercise intervention. All participants provided signed informed consent before participation.

### Experimental procedures

This study was a single-blinded (assessor) parallel-randomized controlled trial. Participants meeting the criteria were randomly assigned to either the experimental group or the control group via a sealed envelope. The study was performed in accordance with the Declaration of Helsinki. Participants in the experimental group participated in a Kinect-based exergaming exercise program for 60 minutes sessions, three times a week for 12 weeks (EXER group). Those in the control group participated in a combined exercise program for 60 minutes sessions, three times a week for 12 weeks (CE group). Exercise training was supervised by an experienced physical therapist in a small group (3–4 people) for both the EXER and CE groups. The outcomes were measured at baseline and after completing the 36 exercise sessions by the same assessor who was blinded to the group assignment.

### Intervention

#### Combined exercise (CE) group

The exercise program followed the guidelines suggested for older populations by the American College of Sports Medicine. This program included 20 minutes of resistance exercises, 20 minutes of aerobic exercises, and 20 minutes of balance exercises.

1. Resistance exercise: The resistance exercises focused on both the upper and lower extremities. Upper extremity resistance training emphasized shoulders (abductors, adductors and rotators), elbows (flexors and extensors), and wrists and fingers (flexors and extensors). Lower extremity resistance training emphasized muscles that are important for balance and gait control. TheraBands were used during exercise, starting with 1.1 kg of resistance (yellow TheraBand) and then gradually increasing to 3.1 kg (green TheraBand). Participants performed 3 sets of 10 to 15 repetitions for each activity with natural breathing during the exercises.

2. Aerobic exercise: The aerobic program was a sequence of whole-body activities, including stepping exercises in the seated and standing positions, stepping on and off of a stool, and standing up from and sitting in a chair. The training intensity was set to 50–75% of the maximal heart rate (220−age), and the perceived exertion rates were recorded during the exercises to modify the intensity. The ideal perceived exertion was a score of 13 to 14 (“somewhat hard”).

3. Balance exercise: The balance exercises combined dynamic and static balance training. The dynamic balance exercises included slow and fast symmetric weight shifting, catching and throwing balls, walking in a straight line, walking sideways, walking backwards, and figure of eight walking. Static balance training included standing with various materials and bases for support, and single leg standing with eyes open and closed.

#### Exergaming (EXER) group

Kinect systems (Microsoft Corporation, Redmond, WA, USA) were used for the exergaming group. The Kinect sensors incorporate infrared light and capture and track changes in limb segment motion during exercise to create a virtual 3D full-body map.

Tano and LongGood software packages, due to their various games, were integrated into the Kinect hardware for the present study. Through avatar technology, a virtual environment was presented on a screen (size: 230 cm × 230 cm) in front of the participants. Participants adjusted their own movements instantaneously according to feedback from the virtual character on the screen. Each session included 20 minutes of Tai-Chi, 20 minutes of resistance and aerobic combination training and 20 minutes of balance training. The details of this program are as follows:

1. Tai-chi exercise: The movement form Tai-chi was adopted from the traditional “Yang style Tai Chi” and was simplified to 24 forms. In this study, the Tai-chi started with a warm-up session, consisting of simple motions to help the elderly relax their muscles and joints. Then, the exercise session emphasized moving the arm and leg in circular motions with low speed and focusing on breathing and muscle coordination. It also emphasized weight shifting and squatting to improve balance, stability, and muscle strength.

2. Resistance and aerobic exercises: The PAPAMAMA program was used for upper/lower extremity strengthening and endurance exercises. The principle of adjusting the intensity followed the same rules as the program used in the CE group.

3. Balance game: In this study, we chose window cleaning, firework hitting, and goldfish grasping games for balance training. When playing these games, participants interacted with the virtual objects on the screen. Participants needed to shift their center of mass with upper/lower extremity movements to reach toward a target in a given location quickly and accurately. In addition, we also chose obstacle crossing and stair stepping games for balance training. During these games, participants needed to lift their legs to cross obstacles or to climb up and down steps at a predetermined speed.

### Outcome measures

#### Primary outcomes

The frailty score, frailty status, frailty reversal rate, and frailty phenotype reversal rate were our primary outcomes. The frailty score has been validated in different populations in Taiwan^39,40^. The frailty score corresponds to the number of the five characteristics of the frail phenotype (unintentional body weight loss, exhaustion, slow walking speed, weakness, and low activity level) an individual displays. One point is allocated if the participant displays one physical characteristic of the frailty phenotype, with the total score ranging from 0 to 5. Frailty status is classified into one of the following 3 statuses: robust (score 0), prefrailty (score 1–2), and frailty (score 3–5), according to the frailty scores. Both the number of participants in each frailty status category and the number of participants displaying each characteristic of the frailty phenotype were calculated. The frailty reversal rate was defined as the fraction of individuals in the frailty status at baseline who improved to the robust or prefrailty status^18^. The equation is expressed as follows: (the number of individuals who were in the frailty status at baseline and improved to the prefrailty or robust status/the total number of individuals in the frailty status at baseline) * 100%.

To investigate the effect of training on each frailty phenotype, the frailty phenotype reversal rate was also defined as the fraction of participants displaying each frailty phenotype at baseline who no longer displayed it after the intervention^18^. The equation is expressed as follows: (the number of participants who displayed each frailty phenotype at baseline and no longer displayed it after intervention/the total number of participants who displayed that frailty phenotype at baseline) *100%.

#### Secondary outcomes

The relationship between physical performance and physical frailty has been established^41^. For example, the Short Physical Performance Battery (SPPB), which is a composite index for testing physical performance, has good concurrent validity and reliability to other measures of physical frailty^42^. Therefore, we chose the following common physical performance indicators as secondary outcomes: (1) back scratch, (2) chair sit and reach, (3) 30-second sit-to-stand, (4) 2-minute step, (5) single leg stance, (6) functional reach, (7) timed up and go, (8) walking speed, and (9) grip strength. The Falls Efficacy Scale International (FES-I), which assesses the fear of fall during daily activities and social-related activities, was also included as a secondary outcome^43^.

### Data analysis

All analyses were performed using SPSS 20.0 software (SPSS Inc. Chicago, IL, USA). Descriptive statistics, including the distribution of variables, were generated for all variables in the form of the mean ± standard deviation or the number (%). Independent t-tests or Chi-square tests were applied to examine the baseline differences in various characteristics between the CE and EXER groups. Two-way analysis of variance (ANOVA) with repeated measures was used to determine the effects of the intervention on frailty scores, physical performance and FES-I. The model effects were group (CE, EXER), time (pre, post), and their interaction. The between-group comparison was the group (CE, EXER), and the within-group comparison was the change over time (pre, post). The post-hoc Tukey test was used for those variables with group × time interaction effects. Chi-square and McNemar tests were used to perform the intergroup and intragroup comparisons of the categorical variables. Effect size (sum of squares between/sum of squares total) was also calculated for the variables with significant group differences. The effect size is presented as the odds ratio (OR) for categorized variables and Cohen’s d for continuous variables.

### Sample size

G*power 3.0 was used to determine the sample size needed for the present study. The frailty score was the primary outcome in this study, and we set the effect size to 0.25, with an alpha level of 5%, power of 80% and a repeated measures ANOVA model. The calculation indicated that 66 participants in total were necessary to achieve sufficient power. However, after recruiting 61 participants, 52 of which completed the study, the effect size η2 was 0.85 and the power was 99% for the 27 participants in the EXER group, and the effect size η2 was 0.66 and the power was 94% for the 25 participants in the CE group. Finally, a total of 52 participants were included in our study because this number was sufficient to achieve statistical power.

## Supplementary information

consort 2010 checklist
